# The Income Elasticities of Food, Calories, and Nutrients in China: A Meta-Analysis

**DOI:** 10.3390/nu14224711

**Published:** 2022-11-08

**Authors:** Jinlu Zhao, Jiaqi Huang, Fengying Nie

**Affiliations:** 1 Agricultural Information Institute of Chinese Academy of Agricultural Sciences, Beijing 100081, China; 2 College of Economics and Management, China Agricultural University, Beijing 100083, China

**Keywords:** food consumption, nutrient, calories, income elasticity, meta-analysis

## Abstract

Estimating food- and nutrient-income elasticities is important for making food and nutrition policies to combat malnutrition. There are many studies that have estimated the relationship between income growth and food/nutrient demand in China, but the results are highly heterogeneous. We conducted a meta-analysis in China to systematically review the elasticity of food, calories, and other nutrients to income. We considered a meta-sample using a collection of 64 primary studies covering 1537 food-income elasticities, 153 nutrient-income elasticities, and 147 calorie-income elasticity estimates. There are significant differences in the size of the income elasticities across food and nutrient groups. We found that food- and calorie-income elasticity appear to decline as per capita income increases, except for vitamin and aquatic products. We also found a publication bias for food and calories, and in particular, the study attributes may be important, as they can influence estimates. Given the limited study on nutrient-income elasticity, understanding the impact of income changes on nutrient intake is an important direction worthy of further research.

## 1. Introduction

Ending poor diets and malnutrition in all its forms is a goal that is intrinsically linked with some of the world’s most pressing challenges. The most recent data show that an unacceptably large number of people are still affected by malnutrition. Globally, of all children under five years of age, one in five is stunted (149.2 million), 45.4 million (6.7%) are wasted, and 38.9 million (5.7%) are overweight [1]. Nutritional burdens trigger some chronic non-communicable diseases and cause enormous health and economic loss in society. Poor diet is the main contributor to nutritional burdens. It is estimated that nearly 690 million people suffer from chronic malnutrition, 3 billion people cannot afford a healthy diet, and 11 million die each year from low-quality diets worldwide [2]. Given the consequences of poor diets, it is particularly important to design evidence-based policies to encourage a healthy diet. Studying people’s food and nutrient demand is essential for policymaking because it explains how economic and policy changes influence people’s demand for food and nutrients.

The coexistence of malnutrition and “hidden hunger” is a common challenge for many emerging economies, including China [3,4]. As the largest developing country, China’s population accounts for about one-fifth of the world [5]. Following the rapid economic growth, the per capita disposable income of Chinese residents saw an average annual growth of 7.2%, in real terms, after adjusting for inflation, from 2011 to 2020. Meanwhile, China’s food consumption pattern has changed from supply-constrained to nutrition-oriented [6]. According to official statistics, China’s share of undernourished people has declined substantially (from 10.6% in 2000–2002 to below 2.5% in 2017–2019) [7]. Yet, there are at least 250 million “hidden hungry (hidden hunger refers to the hunger symptoms of hidden nutritional needs caused by the body’s nutritional imbalance or lack of certain vitamins and essential minerals)” people in China [1], which is characterized by micronutrient deficiency, which is a cause for concern. At the same time, China now finds itself among the nations greatly affected by the growing population of overweight or obese individuals, with an estimated one in six Chinese adults falling into these BMI classifications and childhood and adolescent obesity rates rising over the past several decades [8]. About 70% of chronic non-communicable diseases are linked to hidden hunger and malnutrition [7]. Particularly, the mortality of chronic non-communicable diseases accounted for 85% of all deaths in 2021 and has become a serious concern in China, according to the Report on Chinese Residents’ Chronic Diseases and Nutrition [9]. Given the consequences of poor diet, the size of China’s population, and the rapid growth of per capita income, it is important to study the heterogeneity of Chinese food and nutrient demand with changes in income, providing an evidential basis for designing policies to improve nutrition.

**Figure 1:**
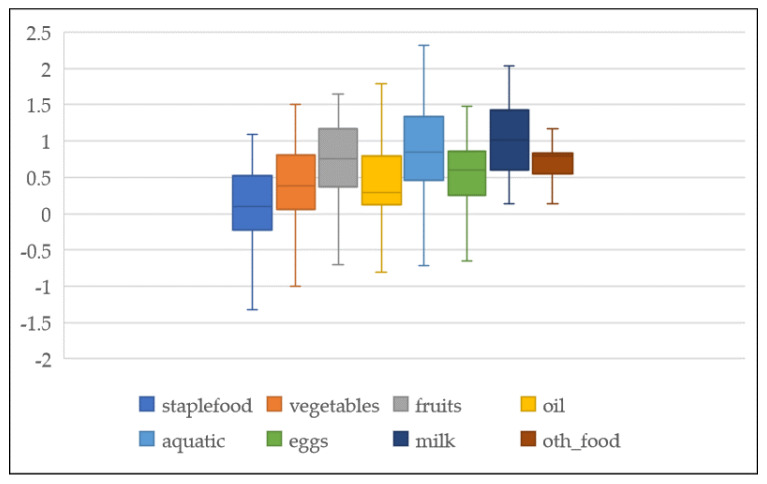
Box plot of food–income elasticities synthesis.

**Figure 2:**
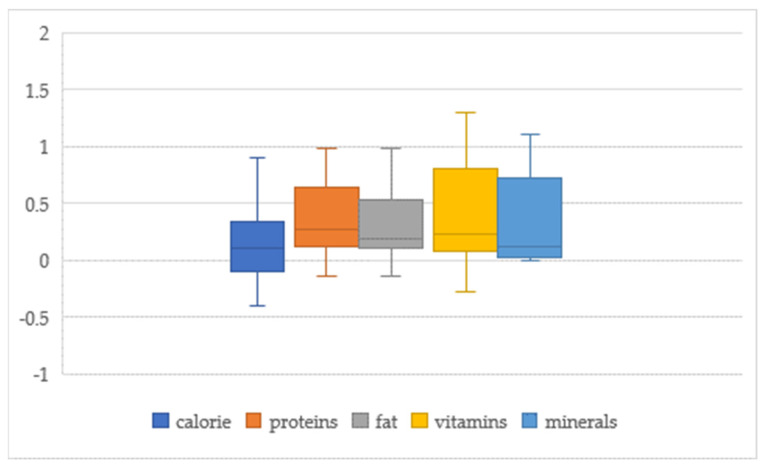
Box plot of calorie-, and nutrient-income elasticities synthesis.

Generally, food demand is income inelastic (elasticities are less than one), reflecting Engel’s law that food budget shares decline when income rises [10]. (Income elasticities of food demand are economic measurements of the responsiveness of food consumption to income for a group of consumers. The income elasticity of food demand measures the percent change in the consumption of total food or a certain food item or group of food items to a percent change in the real income of consumers [11]). However, a series of articles emerged, casting doubt on the role of income. The role of income continues to be interesting, since contrasting results appear throughout the literature [12,13,14]. The food demand–income elasticities estimated in the literature in China showed heterogeneous results. The food demand–income elasticities estimated in the literature ranged from −1.3 to 2.3 (see Figure 1). The income elasticity for staple food reached over 1.0 in a rural study by Han et al. [15]. and Hovhannisyan et al. [16] found that for urban people, the income elasticity for staple food was close to 0, and Carter and Zhong [17] found a negative value. Most studies estimated income elasticities for meat are higher than 0, and some found greater than 1 [6,18,19]. The income elasticities of calories range from −0.4 to 0.9 (see Figure 2). Several authors, such as Liu and Hu [20], Tian [21], and Zhang [22], reported a strong relationship between the level of per capita expenditure and calorie intake. In contrast, Nie and Poza [23] and Chen [24] found that the relationship between household income and calorie intake is not significantly different from zero. Tian and Yu [25] found a nonlinear relationship between nutrition indices and income growth. Households with moderate or higher macronutrient intake tend to decrease their macronutrient intake—especially fat—in rural areas, while in urban areas, the least nourished tend to consume more carbohydrates and proteins [26]. The results are divergent due to different samples, study regions, methods, and other factors.

This study emphasizes the heterogeneity of food-, calorie- and nutrient-income elasticities in China through a meta-analysis approach and builds on previous studies in three ways. First, we analyze a comprehensive set of food and nutrient products at a disaggregate level. Most quantitative reviews of food demand elasticities focused only on calories [27,28] or only on food [4,27,28,29,30,31,32,33,34,35,36,37,38]. Most of these previous meta-analyses focused on a small number of products or products at a more aggregate level than our study. Second, to the best of our knowledge, this is the first review in China that examines the estimates for income elasticity of food, calorie, and nutrients on a comparative basis. Only three meta-analysis studies of food/nutrient demand were published for China: two are on food products [32,39], the other is on calories [29], and none tackles nutrients. The previous meta-analyses of food and nutrient demand exist for other countries or across countries [11,38,40] but not for China, a country with the largest population experiencing a nutrition transition. 

Third, we consider a comprehensive set of potential factors affecting changes in income elasticity, which is linked to the attributes of primary studies. This study updates the estimates of food- and nutrients–income elasticities in China by a systematic approach and provides evidence that may help design food policies in China.

The rest of the paper is structured as follows: Section 2 introduces the data and methods used on income elasticities for food, calorie, and nutrients in China; Section 3 elaborates on the key descriptive statistics of the meta-sample; Section 4 presents an overview of the meta-analysis and model specification estimated in this paper; and Section 5 reports the results, while the discussion section concludes.

## 2. Description of Data

### 2.1. Selection of Primary Studies and Construction of Meta-Sampler

The analysis started with a literature review retrieval to collect food- and nutrient-income elasticities from relevant primary studies. To locate candidate studies, we conducted the initial search across various online databases, including both published literature (journal articles) and “gray” literature (working papers/reports), using the keywords of “China,” “food,” “calorie,” “nutrient,” “demand,” “consumption,” and “income elasticity.” The databases searched were the following: Google, Google Scholar, Web of Science, EconLit, Scopus, the China National Knowledge Infrastructure (CNKI), FAO, and the World Bank. In addition, we conducted an exhaustive search in the references of review studies of China’s food/nutrient demand e.g., [12,32,37,38,39], and we identified 13 new records. 

**Figure 3:**
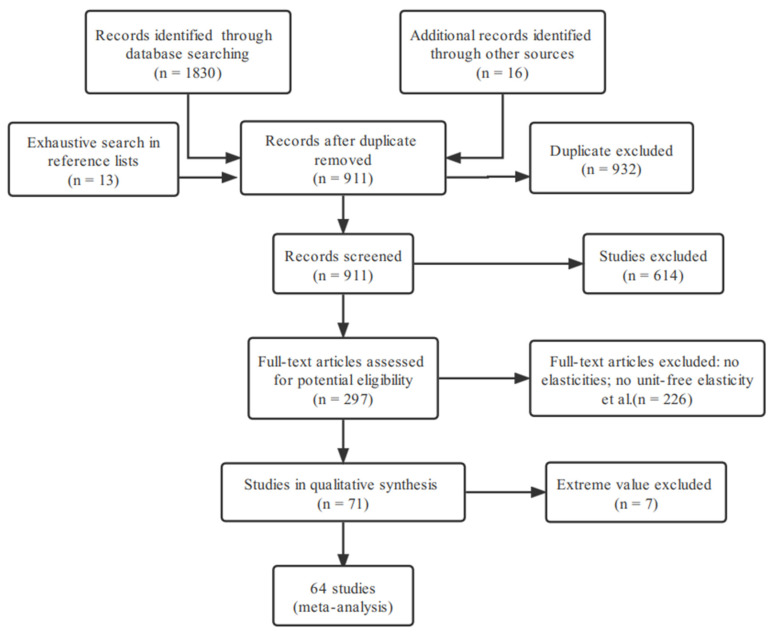
Selection of primary studies and construction of meta-sample.

We based the selection of food-income elasticities on the following criteria. First, after reading and retrieving articles based on the relevance of the abstract to the research objectives, we excluded review articles and repeated literature. This process eventually yielded 898 comparable studies using China data. Second, the primary criteria used in selecting studies for the current analysis was the presence of standard errors or *t*-values for the computed elasticities, and articles did not fulfill this criterion were excluded, reducing the number of articles to 297. Third, to avoid problems of comparability between income-elasticity estimates, we only maintained studies providing unit-free elasticity estimates of food demand concerning income, further reducing the final sample to 71 studies. Fourth, in the case of multiple model estimates for the same dataset, we included only the authors’ recommended model if our determinants do not capture model differences. Finally, we deleted additional observations with food-, calorie-, and nutrient-income elasticities exceeding five standard deviations outside the means to avoid extreme values, resulting in the removal of 7 observations (see Appendix A for a complete presentation of the data and sources). Our final dataset consisted of 64 articles that matched our objective. The study employed primary interest data comprising the dependent variables (meta variables) (income elasticities for food, calories, and nutrients). The resulting samples without outliers provided 1537 food-income elasticity estimates, 153 nutrient-income elasticity estimates, and 147 calorie-income elasticity estimates (where a study produced multiple income elasticities (e.g., for different food/nutrient groups, for urban and rural samples separately, using different estimation models), all estimates were included in the meta-sample, resulting in a total of 147–1537 elasticities) (see Figure 3).

### 2.2. Descriptive Analysis

The explanatory factors can be divided into two categories—contextual and methodological factors—to explain the heterogeneity in income elasticity estimates [41]. The contextual difference may be generated by food or nutrient categories, locations studied, and time periods. (Some meta-analyses use the time period of the research data as an explanatory variable. The reason why we ‘do not use this variable is that it is highly correlated with the explanatory variables in our model (especially the logarithm of per capita income).) Methodological factors may include study design and budgeting, demand models, estimation procedures, and peer-review [36].

Table 1 summarizes the main characteristics of three categories of research samples: food-, calorie-, and nutrient-income elasticities. Table 1 presents the definition of the variables included in this research. Among the three types of research samples, the number of foodstuffs accounted for about 84% of the observations; the number of elasticities attributed to nutrients and calorie contributions was less observed, at about 8% each. Overall, the mean of the income elasticities was highest for food (0.690), with a standard deviation of 0.685, which was followed by nutrients (0.298), with a standard deviation of 0.315, and calories (0.212), with a standard deviation of 0.225. These statistics indicate large variations in income-elasticity estimates for food, calories, and nutrients warrant further investigation.

#### 2.2.1. Product Differences

The income elasticities vary across different types of food and nutrients. The results show considerable differences across previous studies. The food demand income elasticities estimated in the literature ranged from −1.30 to 2.30 (see Figure 1). The main food grouping in this research is the staple food, vegetables and fruit, meat, oil and fat, aquatic products, dairy products, eggs, and other foods (in general, “staple food” is defined as all cereals, wheat, rice, and coarse grains, and “meat” is defined as all meats (including pork, beef, mutton, and poultry)). In theory, the income elasticity for a food group should be a weighted average of products’ income elasticities, as the collected elasticities are taken from different studies at different times and places. The product classifications are consistent with those in the rural and urban household surveys conducted annually by the National Bureau of Statistics of China (NBSC). (Along with the degree of substitution, that is, foods of the same category should have greater substitutability than those of different categories. The “tubers, starch” are summarized as “staple food”, and “sugar and beverages” is merged into “other food” to facilitate meta-regression analysis, with the limited amount of starch, tubers, sugar, and beverages observed in the primary studies.) Food items that make up basic diets, such as staple food, oil, and fruits and vegetables, have lower income elasticities, with a mean elasticity lower than 0.5. However, elasticities are considerably higher for foods sourced from animals. Food groups with higher elasticities would typically supplement basic diet requirements in China (i.e., aquatic products and dairy products) [42], with elasticities ranging between 1.066 and 1.084.

The income elasticities of calories are −0.40 to 0.90, and nutrients are 0.06 to 1.20 (see Figure 2). The mean nutrient elasticities are high, especially for vitamins and fats (Table 1). Nutrients are divided into macronutrients (protein and fat) and micronutrients (vitamins and minerals). The mean elasticity is the lowest for calories (0.212), which is reasonable given that the Chinese have fulfilled the first-order caloric needs [25]. Foods of lower nutritional value and lower-quality diets generally cost less per calorie and tended to be selected by groups of lower socioeconomic status [10]. Conversely, vitamins, protein, and fat-based products are supplementary to the diets of most Chinese households and are seen as high-priced products. The meta-analysis includes dummy variables to control the difference among food/nutrient groups. 

#### 2.2.2. Region-Level Differences

Relatively large differences appear in the magnitude of the income elasticities among regions in China. Data for rural food (0.755) and calorie (0.242) demand indicate that the mean value of income elasticity is larger than urban values (0.560, 0.144). The income elasticity of other nutrients for rural food is lower than for urban. As households meet their first-order caloric needs, they move from cheap calorie-dense staples to more expensive nutrient-dense foods [43]. The reasons for this phenomenon are divided into two aspects. First, urban households have access to a wider variety of food products, and in contrast, rural households have fewer varieties of food because their diets are dominated by households produce, and the local food markets are also underdeveloped [18]. Second, physical activity in urban households is relatively lower than the rural; thus, rural people rely on more calorie requirements, leading to diets comprising more calorie-dense foods [44]. We include a dummy variable for rural data to distinguish between urban and rural areas.

#### 2.2.3. Other Data Differences

To control the difference in data, we set up three dummy variables: (1) how the “income” (total household expenditure or total household income) is measured; (2) whether the data are micro-level survey data or macro summary data; and (3) whether the data are panel data, cross-section, or time-series data.

The primary studies include different definitions of “income” elasticity. The definition of “income” elasticity is the number of demand changes concerning changes in total income (income elasticity) or total household expenditure (expenditure elasticity). (We assume that total household income equals total household expenditures in the long run. However, in the short term, income and expenditure will vary due to savings and borrowing.) There appear to be differences between these two types of studies. For the sake of simplicity, we do not distinguish between income elasticity and total-expenditure elasticity here, while we control for this difference using a dummy variable [41]. In addition, we exclude conditional elasticities calculated by a food-demand model with a function of total food expenditure, or specific food category expenditure, because food expenditure is a family decision variable. If the studies treat total food expenditure or specific food category expenditure as exogenous, concerns arise regarding endogeneity bias [45]. Our sample includes 922 estimated food–income elasticities, and the rest (615 estimates) are total expenditure elasticities. Meanwhile, observations (119) and (113) for calorie/nutrient are total income elasticities, and the rest are expenditure elasticities. The results in Table 1 show that the mean income elasticity of food and nutrients is lower than expenditure elasticity.

Data differences may also lead to systematic differences in elasticity values [12]. Household survey data are generally considered superior to aggregate data; survey data are more consistent with demand theory and may include demographic characteristics, making it possible to examine the heterogeneity of different household preferences [46]. Furthermore, panel data are generally considered superior to cross-sectional data in controlling for unobserved heterogeneity in consumer choice [38,47]. The mean elasticities using panel data are lower than those using other data types (Table 1); thus, we control this data difference analysis using a dummy variable.

#### 2.2.4. Per Capita Income

Low income impacts the quantity and composition of food demand. According to Engel’s Law, as income increases, the proportion of income spent on food decreases. Low-income groups spend nearly half of their budget on food, while high-income groups allocate a small proportion of their income to food. Therefore, low-income groups are more responsive to food prices and income volatility, especially for high-value products [48]. To control for income effects in our meta-analysis, we include the logarithm of per capita income. (If per capita income is reported in the sample literature, we include it. If no income or total expenditure values are reported, we use per capita income values with region in the study year from the National Bureau of Statistics of China to represent it. For studies using panel and time-series data, we take the average of these incomes for the period of the data in the underlying study.)

In addition, Bennett’s Law states that as incomes rise, families change the allocation of their food budgets from starchy staples, cheap sources of calories, to more expensive foods, such as nutrient-rich fruits and animal products. Shifting consumption toward more diversified foods leads to greater nutrient content [49]. Changes in dietary behavior as a function of income may be captured by nonlinear specifications of household food products and nutrient demand functions [23]. The interaction term between the logarithm of per capita income and the different food (or nutrients) is included to explore possible differences.

#### 2.2.5. Modeling and Estimation Differences

We include dummy variables to control four types of models and estimate differences, which are as follows: (1) the type of budget process (multi-stage or single-stage); (2) the type of demand system in the sample study (or only a single equation); (3) whether the demand model is a three-rank Quadratic Almost Ideal Demand System (indicating that the Engel curve in the demand system is nonlinear); and (4) the type of the estimation program in the primary study.

Multi-stage budgeting refers to the sequential allocation of the consumer’s total spending. For example, in a two-stage budgeting model, the consumer decides on the total expenditure of food to be spent in the first stage and then decides on the amount of individual food in the second stage. Multi-stage budgeting requires a weakly separable utility function of consumers among groups of goods [50]; the restriction may affect the estimated value. As seen from Table 1, most studies used single-stage budgeting, and the mean income elasticity of food is 0.721, higher than the mean income elasticity of multi-stage studies (0.540). In contrast, the average value of multi-stage nutrient elasticity was higher than that of a single-stage budget.

Lewbel [51] classifies them among demand systems according to their rank. Demand systems applied nowadays usually have a rank of two or greater to better reflect consumers’ food consumption preference and explain food consumption in responses from different income groups. As Table 1 indicates, most of the meta-samples are two-rank models. For food consumption, the mean elasticity value of the three-rank model is higher than that of the two-rank model. The lack of sample size does not allow comparing the mean value of nutrient elasticity.

Different estimation procedures may have a connection with the estimated elasticities. The Least Squares (LS) is the most popular method, which is used in nearly half of our meta-sample, followed by the method of Quasi-Unrelated Regression (SUR). Other commonly used estimation methods include Maximum Likelihood Estimation (MLE), panel data estimation, and instrumental variables. Among the estimation methods, the mean elastic value estimated by the LS method is lower than that estimated by other methods.

#### 2.2.6. Publication Bias

Publication bias is the phenomenon of easy publication of studies with compelling empirical results of a particular magnitude or statistical significance. Such bias may be caused by several factors, including a tendency for authors, reviewers, and editors to avoid reporting and publishing small and insignificant estimates for statistically significant results. The quality of data and research may also vary among the publication types. The attributes considered include the type of publication used (i.e., published in journals/international organization reports/conference reports/working papers, etc.) and the language of the publication (i.e., published in Chinese/English). 

## 3. Method

Meta-analysis is the quantitative alternative to qualitative reviews of the empirical literature [52]. It is useful in identifying study-specific characteristics that may influence the reported results. There are three main procedures for applying meta-analysis: (i) Funnel Asymmetry Testing (FAT) to test whether publication selection bias influences the sample of estimates; (ii) Precision Effect Testing (PET) to test for a genuine non-zero effect of estimates once the publication bias is accommodated and corrected; and (iii) meta-regression analysis (MRA) to investigate whether study characteristics affected the size of the demand–income relation for food and nutrients.

### 3.1. Funnel Asymmetry Test (FAT) and Precision Effect Test (PET)

Meta-analysis is useful for identifying publication bias and estimating genuine empirical effects beyond publication bias in the effect size. Publication bias may overweigh the real estimated elasticities in the literature, causing the skewed distribution of reported effect size [52,53]. Therefore, it is crucial to identify whether the literature on a given topic suffers from publication selection bias and, if it does, how such bias should be corrected.

A common approach to test the existence of publication bias in the economics literature is the FAT approach [54], which can be specified as follows:

(1) $$\;effect_{ij}=\alpha +\beta_{1}SE_{ij}+\mu_{ij}\;\;\;\;\;\;\;\;\;\;\;\;\;\;\;\;\;\;\;\;$$

where $effect_{ij}$ is the standard effect size from the primary studies, such as the reported elasticity coefficient (i.e., the income elasticity of food/calorie/nutrition) in the primary studies, i denotes the elasticity estimate for the j-th study. $SE_{ij}$ is the standard error in primary studies (in the absence of an estimated standard error, the inverse of the square root of the sample size or the inverse of the square root of the degree of freedom can also be used as a measure of estimation [55]), and $\;\beta_{1}\;$ is statistically significant when the study effect size ($effect_{ij}$) is more likely to correlate with its standard error ($SE_{ij}$). It means publication bias varies with standard error ($SE_{ij}$); large numbers of studies with lower SE values are associated with the significance of $\beta_{1}$, suggesting publication bias.

Stanley [56] proposed a genuine empirical effect beyond the publication bias. Stantely [56,57] suggested applying PET. Based on Equation (1), we consider two sets of covariates: $X_{k}$ is the vector of covariates that explains the heterogeneity associated with estimated elasticity and publication bias; $Z_{m}$ is the vector of covariates to capture the heterogeneity related to the tendency to published estimates. $\beta_{0}$, $\beta_{1}$, $\pi_{k}$, $\delta_{m}$ are estimated parameters, and $\mu_{ij}$ denotes the error term in the regression. The sign and significance of $\beta_{0}$ identify the empirical effect under review of the meta-analysis.

(2) $$effect_{ij}=\beta_{0}+\beta_{1}SE_{ij}+\sum_{k=1}^{K}\pi_{k}X_{k,ij}+\sum_{m=1}^{M}\delta_{m}Z_{m,ij}+\mu_{ij}\;$$

A common problem for the above equation is embedded heteroscedasticity due to the specification of the equation; estimates with small variance are more reliable than those with high variance. Therefore, Equation (2) cannot be estimated directly by the OLS method. Stanley [56] suggests using the inverse of the standard error to correct for heteroscedasticity. Thus, Equation (3) can be re-written as follows:

(3) $$t_{ij}=\beta_{0}\left(\frac{1}{SE_{ij}}\right)+\sum_{k=1}^{K}\pi_{k}(X_{k,ij}/SE_{ij})+\beta_{1}+\sum_{m=1}^{M}\delta_{m}Z_{m,ij}+\mu_{ij}$$

Equation (3) allows us to conduct an FAT to test whether publication selection bias influences the sample of estimates. The larger the deviation between $\beta_{1}$ and 0 ($\beta_{1}\neq 0$), the greater the asymmetry of the effect size reported in the primary studies, thus suggesting publication bias. Equation (3) can also ascertain the genuine empirical effect beyond publication bias through the PET. The $\beta_{0}$ (empirical effect) implies the significance of income on elasticity. If $\beta_{0}$ is statistically significant, we conclude that the impact of income on elasticity is statistically different from 0 in the reviewed studies.

### 3.2. The MRA: Identify Sources of Heterogeneity

The MRA examines the sources of heterogeneity in the population effect size. A typical multivariate MRA is given by the following:

(4) $$E_{ij}=\alpha +\sum_{k=1}^{K}\beta_{k}X_{k,ij}+\mu_{ij};\;\mu_{ij}\sim N\;(0,\;\sigma_{\mu })$$

where $E_{ij}$ is the i-th elasticity of the j-th study (including food, calorie, and nutrients), $X_{k}$ represents the vector of the determined study attribute, and $\alpha ,\;\mu_{ij}$, and $\beta_{k}$ are the estimated parameters. The signs of the reported coefficients ($\beta_{k}$) indicate how a given variable influences changes in the food/nutrient–income elasticity. Thus, a positive sign indicates that the variable positively affects elasticity, and a negative sign implies the opposite effect. The econometric procedure applied to identify the heterogeneity, particularly to reduce publication bias in MRA, is the weighted average of measures using effect size precision (for example, the inverse of the variance of the standard error of the estimated effect size). The standard error in estimating effect size is discounted for small sample studies. Given this, we adopt the method Stanley [55] suggested to estimate Equation (3)’s parameters. This method is a weighted least square model that reduces publication bias in the MRA by using the reciprocal of the square root of the standard error for estimating the weight of the major studies. The variance of each estimate is used as the weight to minimize the variance of the weighted average result.

In light of differences in the primary studies, this article analyzes study characteristics to uncover factors affecting the estimated income elasticities of food, calories, and nutrients. As for the nature of estimate elasticities examined in the primary studies, we grouped three major categories: food-, calorie- and nutrient-income elasticity (the relationship between demand for fats, proteins, minerals, and income) as dependent variables in a series of meta-analyses. The independent variables were stratified based on five dimensions: demand specification, nature of data, estimation technique, published features, and study area. The variables adopted in our study are listed and described in Table 2.

## 4. Results

### 4.1. MST-MRA Results

The initial step for meta-analysis is the FAT and PET, which are obtained through Equation (2). The results presented in Table 3 show that all the coefficients, except nutrient, are statistically different from zero, suggesting that publication bias is only investigated in the food and calorie elasticities used in the analysis. A negative bias occurs in food-income elasticity ($\beta_{1}=-31.39$, *p* < 0.001), indicating that the effect of income on food demand is negatively skewed. In addition, a negative coefficient of $SE_{ij}$ indicates that positive estimates of food–income elasticities appear to be under-reported in the sampled studies. Calorie shows a positive bias $(\beta_{1}=46.62$, *p* < 0.001), indicating the calorie elasticity value favors the positive estimated effect size. In such cases, authors do not report all the results they uncover. Instead, they select results consistent with prior findings or results they believe have a better chance of publication. $\beta_{0}$ reflects the genuine underlying empirical effect after correcting for any publication selection bias. This coefficient is also positive and statistically significant, indicating income’s positive and statistically significant effect on calorie and nutrient intake from the reviewed studies. The Z-variables in Table 3 represent the probability of journal editors’ acceptance of articles for publication. Regarding food elasticity research, the coefficients of journal variable and panel data are positive and significantly different from 0, indicating that articles published in journals and studies using panel data are more likely to exhibit publication bias. Research on calories using micro-survey data is less likely to have publication bias, and single-stage budgeting methods are more prone to publication bias.

### 4.2. Meta-Regression Analyses of Food-Income Elasticities

Table 4 presents the results for all foodstuffs, pooled using Equation (4), and estimated by weighted least squares (WLS) and OLS. Both are similar in the signs and significance levels of the estimated coefficient, indicating that our results are robust. The adjusted R^2^ estimated by WLS is higher than the result of OLS because the WLS was used to reduce the weight of outliers and improve the fitting degree of the model; this is consistent with the assumption that heteroscedasticity exists in the linear meta-regression.

The results show that studies focusing on rural populations estimated significantly higher income elasticities than those for urban or national (rural and urban jointly) populations. This is in line with our expected assumption that urban residents have access to more varieties of foodstuffs. Furthermore, our results on the effect of data attributes on the elasticity were significantly different from those used in primary studies, with lower food demand elasticities for panel data and micro-level survey data. Similar to other studies [27,29], the marginal effect for the total income dummy is negative for food (−0.166). This result may be related to the definition of total income/total expenditures; total income equals total expenditure plus net savings. If the saving rate increases, the estimated elasticities regarding total income may be lower than elasticities for total expenditure [30]. The estimated coefficient for the single-stage dummy is 0.761, indicating the baseline model with multi-rank significantly estimates higher elasticities than single rank.

There is a statistically significant relationship between food elasticities and the log of per capita income, staple food dummy, the aquatic dummy, and the interaction term of per capita income. It confirms differences in income elasticities according to the food group. Elasticities for meat and aquatic products are significantly higher than demand for other food groups; i.e., demand for these foods is most responsive to income changes. Demand for basic foods, such as staple food, is less elastic and thus less responsive to income changes. Concerning the country’s overall income level, our results show that a doubling of per capita income is predicted to lead to a decline of 0.19 in food–income elasticities (−0.27 ×  ln (2) ≈ −0.19). This is consistent with the idea of a saturation point for food consumption and reducing the share of food expenditure as income increases [4]. For staple food, the total marginal effect, including the interaction term, is −0.322–0.270=−0.592, so a doubling of per capita income would lead to a decline of  ln(2)×0.592 ≈ 0.41 in the income elasticity for staple food. With the economic growth, people’s demand for staple foods will become less income-sensitive, indicating that Chinese has met energy needs from starchy staples [6]. For aquatic products, the product of significant public health concern in China, the corresponding increase in income elasticities for aquatic products is about 0.27. Optimizing the consumption structure of animal food and increasing the aquatic products may reduce the risk of heart disease and promote brain and eye health [58]. The increased cost of a healthy diet coincided with higher incomes, so more and more people may seek high-priced aquatic products.

The results show a negative, albeit statistically insignificant, coefficient for the variable representing articles published in journals. Additionally, most of the coefficients for the estimation methods (Model_type, Budget_stage) are not statistically significant. It seems that the estimation procedure and type do not influence much in terms of estimated income elasticities.

### 4.3. Meta-Regression Analyses of Calorie and Nutrient–Income Elasticities

Table 5 presents the results for the calorie and nutrient meta-regression. It explains how publication bias distorts the elasticities—peer-reviewed journals have significantly higher nutrient-income elasticities than working papers/reports. In addition, the marginal effect for Chinese publications is −1.546, which is statistically significant, indicating income elasticity for nutrients in Chinese-language publications is lower than those in English-language publications. The type of data used in primary studies confirms significant differences in nutrient- and calorie-income elasticities. The panel and time-series data appear to have significantly higher estimates for nutrients. However, the calorie–income elasticities of studies that employed panel time-series data were significantly lower in magnitude than those using cross-sectional data. Regarding the functional form of the demand model, the only statistically significant variable is using a demand system for nutrients. Compared with the pragmatic model, the estimated coefficient for the demand system dummy is −1.187, implying that the demand system model tends to yield lower income elasticities for nutrients. As for the multi-stage budgeting model, the only statistically significant result is positive for calories. This result can be related to the fact that a multi-stage budgeting assumption restricts consumption flexibility to adjust to income changes [39].

The nutrient–income elasticity estimated using total income is significantly smaller than the elasticity of total expenditure. There is a significantly negative relationship between income growth and calorie–income elasticity, while the magnitude of the decline in income elasticities is small (about 0.09 in response to a doubling of per capita income,  ln(2)×−0.121 ≈ 0.09). For the income elasticity of nutrient products, the vitamins dummy and the interaction term between the log of per capita income and the vitamins dummy are statistically significant. The income elasticities for nutrients (as a whole), fat, and minerals do not change significantly with income growth. However, for vitamins, the total marginal effect, including the interaction term, is 0.169−0.0122=0.157, so a doubling of per capita income would lead to an increase of ln(2)×0.157 ≈ 0.11 in the income elasticity for vitamins. It appears that the intake of vitamins will exhibit greater decreases in the face of economic downturns than other nutrients. In particular, results here suggest that as countries become richer, not only are calorie intakes response to income change on a gentle trajectory, but that the impact on nutrient intake is likely to be small.

## 5. Discussion and Conclusions

This study aims to better understand the relationship between income level and the demand for different foods, nutrients, and calories in China. This will help in understanding what domestic policies can be used in the fight against malnutrition. A significant contribution of the study is creating a database of food-income elasticities that can identify the factors underlying differences in estimates in China. A meta-sample of income elasticities for China was built, drawn from 64 primary studies, covering 1537 food-income elasticities for eight groups of food (Staple food, Vegetables and fruit, Meat, Oil and fat, Dairy, Aquatic products, Eggs, and Other food), 153 nutrient–income elasticities for three types of nutrients (fat, vitamin, and minerals), and 147 calorie–income elasticity estimates. The variables capturing a set of study-specific attributes identified important factors associated with the variation in primary studies to explain the heterogeneity across income elasticities.

On average, income elasticities in China are positive across all food categories. Thus, income is a potential key determinant of food demand. According to the meta-analysis results, elasticities for meat and aquatic products are significantly higher than demand for other food, while staple food is less elastic. Moreover, there is a significant negative relationship between income growth and the size of food–income elasticities, that is, the food–income elasticities decline with income growth. This relation also holds for staple food. One exception is the aquatic products; we find a positive relationship between income growth and the magnitude of income elasticity of aquatic products. A possible explanation is that the saturation point of staple food consumption has been reached due to increased incomes. Chinese people may shift consumption toward more diversified and often more expensive foods with higher nutrient content [59]. These results suggest that income growth may align with a beneficial shift in the nutritional status of diets to reduce the risk of heart and brain disease [58].

Concerning nutrients income elasticities, we found the mean income elasticity is higher for fat (0.324), vitamin (0.304), and protein (0.303) than for minerals (0.248) and calories (0.212). The impact of income on calorie and nutrient intake is likely to be small, as some studies have concluded [38,60]. Knowing how calorie and nutrient elasticities change with income becomes necessary in light of nutrition-related chronic diseases. There is a significantly negative relationship between income growth and calorie–income elasticity size, while the predicted decline is relatively small (0.09 in response to a doubling in per capita income). However, given that China’s per capita income is growing by 7.2%, the results suggest that the sensitivity of caloric demand to income will be constant though incomes rising. Thus, for countries that are already consuming well beyond the recommended calorie levels, further increases in income will lead to an even larger consumption of calories. Thus, policy-makers still should primarily focus on the alleviation of weight-related problems, with one in six Chinese adults falling into obesity [6,8]. As for nutrients, only the income elasticity for vitamins may have a gentle increase with income growth, while the impact on other nutrients is not significant. The effect of nutrients on the chronic disease has also been documented. Vitamin D supplementation has been associated with reduced mortality. Zinc deficiency has caused children growth retardation. Vitamin A, Vitamin E and total antioxidant capacity supplementation have had a protective effect on metabolic syndrome [61]. Regarding the steadily increasing incidence of nutrition-related chronic diseases [1], economic growth accompanied by nutrition transition patterns tends to evoke health problems. Some studies suggest that good nutrition is a driver of economic growth. Therefore, development policies should be geared specifically toward reducing chronic malnutrition to spur economic growth rather than focusing on economic growth to spur good nutrition [62,63]. Policy-makers should continue to monitor the evolution of demand for these nutrients to ensure people’s health, particularly given the sheer size of the population and the relatively tight nutrition situation in China.

Our results prove the relationships between regions and food-, calorie-, and nutrient-income elasticity. Regarding food, we found that rural people are more sensitive to income and price change, which is consistent with earlier findings in the literature [63,64]. We did not find significant effects of urban and rural differences concerning calories and nutrients.

As for the role of data and methodology, we found that the food elasticity is higher when the primary studies substitute household expenditures for income, similar to [29]. In addition, we found publication bias in studies on food and calorie elasticities; the elasticity values reported in journals were lower than those in reports or working papers, which is in line with the previous studies [27,28]. Specifically, we found that income elasticity for nutrients in Chinese-language publications tends to be lower than in English-language publications.

Overall, the results suggest that development strategies to improve economic growth may improve diet quality but may be insufficient to improve nutrient intake. This study’s findings also suggest that heterogeneity in food-, calories-, and nutrients-income demand elasticities in China are mainly due to contextual characteristics and methodological factors. Notably, we also find publication bias. It is crucial to be aware of these causes of heterogeneity and provide reliable income elasticity estimates to improve food demand projections and design effective food and nutrition policies in China. This study’s main limitation is that the number of examined articles may be insufficient; only a few studies investigate nutrient–income elasticity in China, much less than food demand. Due to the nature of our study, relying on a more extensive study set does not seem feasible for the current meta-analysis. Understanding the impact of income changes on nutrient intake is still an important direction worthy of further research.

## Figures and Tables

**Table 1:** Summary statistics of meta-sample.

Income Elasticities	Food-Income Elasticities			Calorie-Income Elasticities			Nutrient-Income Elasticities		
	Number	Mean	Std. Dev.	Number	Mean	Std. Dev.	Number	Mean	Std. Dev.
***Total***	1537	0.690	0.685	147	0.212	0.325	153	0.298	0.315
***Published features***									
English	385	0.724	0.362	70	0.187	0.284	69	0.249	0.290
Chinese	1152	0.678	0.766	77	0.233	0.367	84	0.342	0.345
Journal	1291	0.695	0.703	105	0.203	0.342	107	0.333	0.334
Other	246	0.662	0.593	42	0.288	0.293	46	0.281	0.304
***Area***									
Rural	676	0.755	0.579	49	0.242	0.267	46	0.225	0.184
Urban/Nation	861	0.560	0.615	56	0.144	0.377	61	0.317	0.340
***Data***									
Macro-aggregate	553	0.722	0.804	63	0.178	0.269	61	0.270	0.229
Micro-survey	984	0.654	0.568	84	0.265	0.368	92	0.350	0.371
Panel	953	0.439	0.672	70	0.137	0.181	77	0.166	0.165
Time series	96	0.567	0.447	17	0.206	0.104	23	0.244	0.029
Other data	488	0.680	0.491	60	0.324	0.351	54	0.526	0.193
***Income measure***									
Expenditure	615	0.924	0.376	28	0.194	0.149	23	0.386	0.384
Income	922	0.531	0.796	119	0.216	0.350	130	0.288	0.318
***Type of estimator***									
FE/RE	104	0.878	0.744	14	0.100	0.001	23	0.042	0.030
IV/GMM	64	0.923	0.087	28	0.527	0.215	31	0.467	0.257
LS	973	0.493	0.608	84	0.113	0.280	84	0.205	0.275
MLE	64	0.937	0.221	7	0.985	n/a	8	0.903	n/a
SUR	332	0.908	0.488						
***Type of model***									
Demand system	1351	0.777	0.659	70	0.280	0.362	77	0.387	0.392
Single equation	186	0.059	0.544	77	0.157	0.297	77	0.249	0.219
***Type of budget***									
Multi	264	0.540	0.686	28	0.346	0.379	23	0.601	0.544
Single	1273	0.721	0.685	119	0.179	0.314	130	0.263	0.283
Model_rank 3	326	1.062	0.482		n/a	n/a	8	0.114	0.000
Model_rank 2	1025	0.590	0.699	147	0.227	0.325	145	0.317	0.321
***Type of food***									
Staple food	242	0.146	0.807	-	-	-	-	-	-
Vegetables and fruit	245	0.434	1.096	-	-	-	-	-	-
Meat	270	0.865	1.099	-	-	-	-	-	-
Oil and fat	151	0.492	0.673	-	-	-	-	-	-
Aquatic products	215	1.066	1.078	-	-	-	-	-	-
Eggs	220	0.744	1.894	-	-	-	-	-	-
Dairy	91	1.084	0.613	-	-	-	-	-	-
Other food	105	1.102	0.759	-	-	-	-	-	-
***Type of nutrients***									
Protein	-	-	-	-	-	-	20	0.303	0.296
Fat	-	-	-	-	-	-	16	0.324	0.326
Vitamin	-	-	-	-	-	-	8	0.304	0.235
Minerals	-	-	-	-	-	-	6	0.248	0.426

Note: n/a: not applicable. Std. Dev.: Standard Deviation. FE/RE: Fixed Effects/Random Effects. IV/GMM: Instrumental variables estimation/Generalized Methods of Moments. LS: Least Squares. MLE: Maximum Likelihood Estimate. SUR: Quasi-Unrelated Regression.

**Table 2:** Description of the variables.

Category	Variables	Description
Published features	Pub_journal	Dummy variable: 1 = peer-reviewed journal, 0 = report/working paper
	Pub_chinese	Dummy variable: 1 = Chinese, 0 = English
Study area	H_region	Dummy variable: 1 = rural, 0 = other (including urban and nation)
Income measure	H_income	Dummy variable: 1 = total income, 0 = total expenditure
Data	D_micro	Dummy variable: 1 = micro-level survey data, 0 = macro-level aggregate data
	D_panel	Dummy variable: 1 = panel, 0 = others
	D_time series	Dummy variable: 1 = time series, 0 = others
Model and method	Model_type Budget_stage	Dummy variable: 1 = demand system, 0 = pragmatic model Dummy variable: 1 = single-stage, 0 = multi-stage
	Model_rank	Dummy variable: 1 = model_rank 3, 0 = model_ rank 2
Per capita income level	lncome	Continuous variable: Log of per-capita annual disposable income
Food group	Staple food, Vegetables and fruit, Meat, Oil and fat, Dairy, Aquatic products, Eggs, Other food	Dummy variable: 1 = * food, 0 = others
Nutrient group	Protein, Fat, Vitamin, Minerals	Dummy variable: 1 = * nutrient, 0 = others
Interaction *	* lnincome	Interactions between individual food or nutrient dummy variables (represented by *) and logarithms of per capita income

Note: Individual food or nutrient dummy variables (represented by *)

**Table 3:** The results of FAT-PET.

Variables	Food	Calorie	Nutrition
1/SE (empirical effect-$\beta_{0}$)	0.384	0.417 *	0.661 *
	(1.64)	(1.89)	(1.85)
X-variables			
H_income/SE	−0.116	−0.230	−0.406
	(−1.05)	(−0.99)	(−1.18)
Model_type/SE	0.767 ***	−0.240	−0.284
	(3.15)	(−1.50)	(−0.89)
Model_rank/SE	0.0695		
	(0.83)		
Z−variables			
Pub_journal	22.55 ***	−7.183	0.327
	(4.90)	(−0.64)	(0.02)
H_region	4.195	−8.175	−6.106
	(1.34)	(−0.70)	(−0.40)
D_micro	1.942	−21.98 *	−11.42
	(0.86)	(−1.97)	(−0.40)
D_panel	4.690 *	−11.53	−13.29
D_time series	(1.99)	(−0.90)	(−0.75)
	−3.443	−27.91	−2.424
	(−0.90)	(−1.64)	(−0.10)
Budget_stage	0.532	32.28 **	17.26
	(0.16)	(2.57)	(0.90)
Constant (publication bias−$\beta_{1}$)	−31.39 ***	46.62 ***	33.91
	(−5.78)	(3.59)	(1.25)
Number of observations	1537	147	153
R^2^	0.997	0.821	0.577

Note: *t*-values are given in parenthesis. * *t* < 0.1, ** *t* < 0.05, *** *t* < 0.01.

**Table 4:** Results from the meta-regressions of food–income elasticities.

	Variables	OLS	WLS
Publication	Pub_journal (1 = peer-reviewed journal, 0 = report/working paper)	−0.0567	−0.0793
		(−0.21)	(−0.41)
	Pub_chinese (1 = Chinese, 0 = English)	−0.129	0.0570
		(−0.57)	(0.37)
Study area	H_region(1 = rural, 0 = other)	0.311 *	0.745 ***
		(1.98)	(5.15)
Data	D_micro (1 = survey data, 0 = aggregate data)	−0.296	−0.312 **
		(−1.36)	(−2.11)
	D_panel (1 = panel, 0 = others)	−0.109	−0.214 *
		(−0.69)	(−1.75)
	D_time series (1 = time series, 0 = others)	−0.219	0.559
		(−0.48)	(0.45)
Income measure	H_income (1 = total income, 0 = total expenditure)	−0.354 **	−0.166 *
		(−2.03)	(−1.69)
Model and method	Model_type (1 = demand system, 0 = pragmatic model)	0.185	0.382
		(0.69)	(1.38)
	Budget_stage (1 = single-stage, 0 = multi-stage e)	−0.0515	−0.0788
		(−0.25)	(−0.51)
	Model_rank (1 = model_rank 3, 0 = model_ rank 2)	0.490 **	0.761 ***
		(2.20)	(4.17)
Types of food	Staple food	−1.833	−1.422 *
		(−0.47)	(−1.98)
	Vegetables and fruit	4.185	5.473
		(1.65)	(1.59)
	Oil and fat	−1.783	−0.252
		(−0.91)	(−0.13)
	Dairy	0.701	−2.107
		(0.23)	(−0.86)
	Aquatic products	−2.179	3.009 ***
		(−1.35)	(−2.99)
	Eggs	−1.540	0.883
		(−0.89)	(0.63)
	Other food	−1.085	−3.526 *
		(−0.58)	(−1.76)
Income	lnincome	−0.206	−0.270 *
		(−0.69)	(−1.40)
Interaction *	Staple food * lnincome	−0.231	−0.322 *
		(0.53)	(1.95)
	Vegetables & fruit * lnincome	−0.484	−0.552
		(−1.67)	(−1.48)
	Oil and fat * lnincome	0.239	0.0574
		(1.06)	(0.25)
	Dairy product * lnincome	−0.100	0.217
		(−0.30)	(0.79)
	Aquatic products * lnincome	0.240	0.359 ***
		(1.23)	(2.80)
	Eggs * lnincome	0.231	−0.0395
		(1.09)	(−0.22)
	Other food * lnincome	0.148	0.414 *
		(0.68)	(1.84)
	Constant	2.348	5.371
		(0.60)	(1.23)
	Number of observations	1537	1516
	Number of studies	58	57
	R^2^	0.540	0.746

Note: *t*-values are given in parenthesis. * *t* < 0.1, ** *t* < 0.05, *** *t* < 0.01.

**Table 5:** Results from the meta-regressions of calorie-, nutrient–income elasticities.

	Variables	Calorie	Nutrient
Publication	Pub_journal	0.107	−1.546 *
		(0.50)	(−11.48)
	Pub_chinese	−0.0428	−0.711 **
		(−0.26)	(−21.56)
Study area	H_region	0.128	0.0553
		(0.87)	(3.45)
Data	D_micro	−0.0791	0.171
		(−0.33)	(2.46)
	D_panel	−0.268	1.012 **
	D_time series	(−0.69)	(17.45)
		−0.946 *	1.183 **
		(−1.86)	(13.58)
Income measure	Income	−0.247	1.176 **
		(−0.99)	(−10.64)
Model and method	Model_type	−0.478	−1.187 **
		(−1.34)	(−35.57)
	Budget_stage	0.844 ***	0.623
		(3.73)	(6.14)
Types of nutrients	Fat		1.526
			(5.26)
	Vitamin		−1.216 *
			(11.57)
	Minerals		6.596
			(3.25)
Income	lnincome	−0.121 *	−0.0122
		(1.95)	(0.60)
* lnincome	Fat * lnincome		−0.0796
			(−2.36)
	Vitamin * lnincome		0.169 **
			(−54.04)
	Minerals * lnincome		−0.757
			(−3.58)
	Constant		2.261 **
			(15.00)
	Number of observations	147	153
	Number of studies	20	19
	R^2^	0.942	0.989

Note: *t*-values are given in parenthesis. * *t* < 0.1, ** *t* < 0.05, *** *t* < 0.01.

## Data Availability

The dataset used during the current study are available from the corresponding author on reasonable request.

## Appendix A

**Table A1:** List of Primary Studies.

Author	Journal	Time	Title	Area	Elasticity Value
Su Chang	Master’s thesis,	2010	Effects of Economic Factors on Dietary Structure and Nutritional Status of Adult Residents in China—A Case Study of Nine Provinces (1991–2006)	Urban	staple food −0.046 oil and fat 0.015 meat 0.328 aquatic products 0.483 eggs 0.056
Yu Wen, Xu Shiwei, Zhang Yumei, Li Zhiqiang, Li Zhemin	*Chinese food and nutrition*	2012	Study on food consumption and nutritional needs of rural households in China—based on cross-sectional data analysis of rural households	Rural	staple food 1.000 vegetables and fruit 0.989 oil and fat 0.996 meat 0.987 other food 0.990 calorie 0.362 protein 0.336 fat 0.533 minerals 1.102
Zhang Yumei, Yu Wen, Li Zhiqiang	*Journal of Jiangxi Agricultural University*	2012	Study on the elasticity of food consumption demand of rural residents in China	Rural	staple food 0.290 oil and fat 0.100 meat 1.810 aquatic products 2.010 eggs 0.540
Feng Zhiming, Steenfeng	*Resource science*	2006	In the past 20 years, the changes of food consumption and the evaluation of dietary nutrition in China	Nation	staple food −0.364 meat 0.985 aquatic products 0.863 eggs 1.062
Yu Wen, Xu Shiwei		2012	Analysis of food consumption by rural residents in China in 2012	Rural	staple food 1.00 vegetables and fruit 0.089 oil and fat 0.996 meat 0.987 other food 0.990
Liu Weiwen, Ye Songqi, Liu Zhixiong	*China market*	2015	The characteristics and differences of food consumption structure of urban and rural residents-take Beijing as an example	Rural	staple food 0.850 meat 0.267 eggs 0.285 other food 1.000
Li Huishan, Xu Shiwei, Kong Fantao	*Consumer economy*	2015	A study on food consumption of urban residents in China based on income stratification	Urban	staple food 0.017 oil and fat 0.270 meat 1.027 aquatic products 1.803 eggs 0.813 dairy products 2.033 other food 1.567
Sun Guofeng, Liu Weijin, Gao Yanchun	*Journal of Huazhong Agricultural University (Social Science Edition)*	2002	Empirical analysis of the food consumption structure of urban residents in Jiangsu Province	Urban	staple food 0.329 oil and fat 0.343 meat 0.612 aquatic products 0.943 eggs 0.582 dairy products 1.574 other food 1.280
Chang Xiangyang, Li Aiping	*Journal of Nanjing Agricultural University (Social Science Edition)*	2006	Study on food consumption demand of rural residents in Jiangsu Province	Rural	staple food 0.072 vegetables and fruit 0.387 oil and fat 0.150 meat 0.536 aquatic products 0.855 other food 0.771
Li Xiaojun, Li Ninghui	*Statistical research*	2005	Measurement and analysis of food consumption behavior of rural residents in the main food producing area	Rural	staple food 0.886 meat 1.058 aquatic products 0.879 eggs 0.760
Xiaoli Zuo, Zhang Guangsheng	*Journal of Shenyang Agricultural University (Social Science Edition)*	2011	Study on animal food consumption of urban and rural residents in Liaoning Province	Urban	meat 1.004 aquatic products 1.003 eggs 1.168
Zhou Jinchun	*Chinese rural observation*	2006	Study on the AIDS model of food consumption in rural residents	Rural	staple food 0.560 vegetables and fruit 1.804 oil and fat 0.219 meat 1.159 aquatic products 2.512 eggs 0.835 dairy products 2.190 other food 2.275
Huang Jixuan	*Chinese social sciences*	1999	Social development, urbanization and food consumption	Urban	staple food 0.497 vegetables and fruit 1.059 meat 0.651 aquatic products 1.460 other food 0.960
Cheng Lichao	*Southern economy*	2009	Intergenerational Differences in Food Consumption: An Empirical Study Based on China Health and Nutrition Survey	Urban	staple food 0.029 vegetables and fruit 0.004 meat 0.053 eggs 0.030
Wang Zhigang, Xu Qianjun	*Quantitative Economics, Technology and Economics Research*	2012	Explore the transformation law of the food consumption structure of rural- the application of the LA/AIDS model embedded in the time path	Rural	staple food 0.416 meat 0.457 eggs 0.777 other food 0.123
Liu Xiumei, Qin Fu	*Agricultural technology economy*	2005	Study on animal food consumption of urban and rural residents in China	Nation	meat 0.977 eggs 0.658
Huang Xintian, Yifahai	*Consumer economy*	1999	Model analysis of the changing trend of the main food consumption structure of urban residents in China	Urban	staple food 0.258 vegetables and fruit 0.520 meat 0.402 aquatic products 0.438 eggs 0.421
Xu Shiwei, Yu Wen, Wang Wei	*Journal of Nutrition*	2015	Analysis of rural household food needs in rural China	Rural	staple food 1.000 vegetables and fruit 0.989 oil and fat 0.996 meat 0.987 other food 0.990
Yu Wen, Liu Hong, Wang Dongjie, Wang Wei	*Chinese food and nutrition*	2012	Study on the consumption of rations for Chinese residents	Rural	staple food −0.028
Li Hao	Master’s thesis, Chinese Academy of Agricultural Sciences	2007	Study on food consumption and nutrition of poor residents in urban areas: an empirical analysis from six counties in Hunan Province	Urban	staple food 0.102 vegetables and fruit 0.255 oil and fat 0.106 meat 0.104 aquatic products 0.149 eggs 0.143
Li Aiping	Master’s thesis, Nanjing Agricultural University	2007	Study on food consumption of residents in rural Jiangsu Province	Rural	staple food 0.079 vegetables and fruit 0.372 oil and fat 0.185 meat 0.501 aquatic products 0.816 other food 0.530
Sun Feifei	Master’s thesis, Nanjing Agricultural University	2012	Study on food consumption and nutrition of residents in rural Jiangsu Province	Rural	staple food 0.335 vegetables and fruit 0.307 oil and fat 0.228 meat 0.281 aquatic products 0.268 eggs 0.250 dairy products 0.140
Liang Fan	Master’s thesis, Northwestern University of Agriculture, Forestry and Technology	2014	Analysis of changes in food expenditure and nutritional structure of urban residents in Shaanxi Province	Urban	staple food 0.165 vegetables and fruit 0.822 oil and fat 0.177 meat 0.607 aquatic products 0.952 eggs 0.539 dairy products 1.172
Bi Jieying	Master’s thesis	2010	Study on food consumption among Chinese rural poor	Rural	staple food 0.310 vegetables and fruit 0.760 oil and fat 0.820 meat 0.030 aquatic products 2.630 eggs 1.140 dairy products 2.560 other food 0.610
Zhang Pinying	*Statistics and decision-making*	2013	A study on the heterogeneity of food demand of urban residents in China based on THEIDS model	Urban	staple food 0.930 vegetables and fruit 1.141 meat 1.088 aquatic products 1.054 eggs 0.611 dairy products 0.921 other food 0.836
Wu Wei, Chen Yongfu, Yu Law Steady	*Chinese rural observation*	2012	Analysis of food consumption behavior of urban residents in Guangdong Province based on the income stratification QUADDS model	Urban	staple food 0.731 vegetables and fruit 0.992 oil and fat 1.026 meat 1.060 eggs 0.767 dairy products 1.454 other food 0.864
Zhang Mingyang, Zhang Chess	*Consumer economy*	2015	Study on the transformation of the food consumption structure of rural residents—an application of the QUADS model that addresses expenditure constraints and embeds demographic characteristics	Rural	oil and fat 1.086 meat 1.086 aquatic products 0.841 eggs 0.797
Chen Chao, Zhang Mingyang	*Nanjing Social Sciences*	2013	Study on the impact of the implementation of gmed economy food policy on the change of food consumption structure of urban residents	Urban	staple food 3.870 vegetables and fruit 0.790 oil and fat 1.790 meat 0.480 aquatic products 1.040 eggs 6.690 other food 2.400
Gao Shuai	Doctoral thesis	2013	Food safety research for farmers in poor areas	Rural	staple food −0.571 vegetables and fruit 2.973 oil and fat 3.920 meat 5.465 aquatic products 1.322 eggs 1.056
Huang Jiaxuan, Yin Fengying, Yu Yanzhang	*Consumer economy*	2016	Study on the food consumption demand of farmers in poor counties	Rural	staple food 0.847 vegetables and fruit 1.114 oil and fat 0.764 meat 1.623 aquatic products 1.365 eggs 1.192 dairy products 0.885
Zhang Xuemei	Master’s thesis	2013	Study on food consumption and nutrition of poor residents in rural China under the background of rising agricultural prices	Rural	staple food –0.040 vegetables and fruit 0.269 oil and fat 0.126 meat 0.345 aquatic products 0.447 dairy products 1.222 other food 0.381 calorie 0.026 protein 0.03 fat 0.162
Han Yuru, Chen Yongfu	*Rural economy*	2016	Based on the income stratified QUADDS model, this paper makes an empirical analysis of the factors influencing food consumption in migrant workers’ families	Rural	staple food 1.073 vegetables and fruit 1.180 oil and fat 1.077 meat 0.925
Zhang Yumei, Xu Xin, Li Zhiqiang	*Consumer economy*	2012	Dynamic analysis of food consumption demand—based on rural household survey data	Rural	staple food 0.275 vegetables and fruit 0.345 oil and fat 0.375 meat 0.315 aquatic products 0.465
				Urban	staple food −1.425 vegetables and fruit −0.750 oil and fat −0.500 meat −0.750 aquatic products −0.950
Zheng Zhihao, Gao Ying, Zhao Yinxuan	*Economics* (Quarterly)	2015	The influence of income growth on the consumption pattern of food in urban areas	Urban	staple food 0.322 vegetables and fruit 0.441 oil and fat 0.277 aquatic products 0.784 eggs 0.487 dairy products 0.908
Huang Jiaxuan	Master’s thesis	2014	Study on food consumption and nutrition of farmers in poor areas of western China	Rural	staple food 0.600 vegetables and fruit 1.159 oil and fat 0.800 meat 1.626 eggs 1.293
Zhang Mingyang, Zhang Qi	*Consumer economy*	2015	Study on the transformation of food consumption structure in rural residents—an application of the QUAIDS model that addresses expenditure constraints and embeds demographic characteristics	Rural	oil and fat 1.086 aquatic products 0.841 eggs 0.797
Li Dongsheng, Yang Yiqun	*Journal of Wuhan University of Technology*	2001	ELES model of food consumption needs of urban and rural residents	Urban	staple food 0.133 vegetables and fruit 0.312 oil and fat 0.676 meat 0.111 eggs 0.482 other food 0.592
				Rural	staple food 0.166 vegetables and fruit 0.405 oil and fat 0.574 meat 0.358 eggs 0.679 other food 0.619
Yuan Mengxuan, Li Xiaoyun, Huang Malan	*Rural economy and technology*	2019	Analysis of nutritional income elasticity of urban and rural residents in Hubei Province—consumption and nutritional changes of staple foods	Rural	protein −0.280 fat 0.265
				Urban	protein −0.133 fat 0.223
Han Xiao, Qi Weitian, Wang Xinghua	*Journal of Beijing University of Aeronautics and Astronautics (Social Science Edition)*	2019	Impact of income increases of urban residents’ on food consumption patterns: based on two-phase EASI model	Urban	staple food −0.673 vegetables and fruit 0.697 oil and fat 0.078 meat 1.457 aquatic products 4.451 eggs 0.368
Wang Jun, Zhuang Tianhui, Chen Xue	*Journal of Sichuan Agricultural University*	2017	Analysis of the consumption of livestock products in Xinjiang	Nation	staple food −0.140 meat 0.375 aquatic products 0.574 eggs 0.320 dairy products 0.274
Mu Yueying, Haosan Sakahara, Minxin Matsuda	*Economic problems*	2001	Analysis of THEDS model of China’s urban and rural consumer demand system	Nation	staple food 0.240 meat 0.275 vegetables and fruit 0.397 aquatic products 0.574 eggs 0.620 dairy products 0.574
Li Guojing, Chen Yongfu, Yang Chunhua	*Agricultural technology economy*	2018	Income growth, difference in household registration and nutritional consumption—based on research on migrant workers’ families entering the city	Rural	calorie 0.593 protein 0.610 fat 0.533
Zhang Chewei, Cai Fang	*Economics* (Quarterly)	2002	China’s poor rural food demand and nutritional elasticity	Rural	calorie 0.145
Ye Hui, Wang Yapeng	*Agricultural technology economy*	2007	Analysis of the impact of changes in staple food prices and income on national nutrition	Nation	calorie −0.124 protein −0.138 fat −0.143
Yuan Mengxuan	Master’s thesis	2017	Study on consumption and nutritional elasticity of staple foods in Hubei Province	Urban	calorie 0.175 protein 0.265 fat 0.147 vitamin −0.280
				Rural	calorie 0.221 protein 0.223 fat 0.208 vitamin −0.133
Dong Guoxin, Lu Wencheng	*Forum on Statistics and Information*	2009	Analysis of AIDS model of food consumption among Chinese residents—citing western urban areas as an example	Urban	staple food 0.080 vegetables and fruit 0.807 oil and fat 1.652 meat 0.523 eggs 1.714 other food 1.254
Li Guojing, Chen Yongfu	*Southern economy*	2018	Income level, aging and nutritional intake—based on data on urban households in Guangdong Province	Urban	protein 0.749 fat 0.678 vitamin 0.661
Fengying Nie, Jiaqi Huang, and Jieying Bi	*Proceedings of 2013 World Agricultural Outlook Conference*	2013	Food Consumption of Households in Poverty-Stricken Areas of West China: The Case of Shaanxi, Yunnan, and Guizhou	Rural	staple food 0.649 vegetables and fruit 0.870 oil and fat 1.279 meat 0.769 eggs 1.739 other food 1.165
Zhihao Zheng and Shida Rastegari Henneberry	*Journal of Agricultural and Resource Economics*	2010	The Impact of Changes in Income Distribution on Current and Future Food Demand in Urban China	Urban	staple food 0.136 vegetables and fruit 0.249 oil and fat 0.356 meat 0.318 eggs 0.458 dairy products 0.332 other food 0.368
Brian W. Goulda, Hector J. Villarrealb	*Agricultural Economics*	2006	An assessment of the current structure of food demand in urban China	Urban	staple food 0.955 vegetables and fruit 0.950 oil and fat 0.850 meat 1.340 eggs 1.400 dairy products 0.620 other food 0.960
X.M. Gao, Eric J. Wailes, and Gail L. Cramer	*American Journal of Agricultural Economics*	1996	A Two-Stage Rural Household Demand Analysis: Microdata Evidence from Jiangsu Province, China	Rural	staple food 0.516 vegetables and fruit 1.258 oil and fat 0.722 meat 0.722 eggs 0.892 calorie 0.787
Shenggen Fan, Gail Cramer, Eric Wailes	*Agricultural Economics*	1994	Food demand in rural China: evidence from rural household survey	Rural	staple food 0.303 vegetables and fruit 1.097 meat 1.688
Fred Gale and Kuo Huang	Economic Research Report	2009	Demand for Food Quantity and Quality in China	Rural	staple food 0.087 vegetables and fruit 0.263 oil and fat 0.453 meat 0.293 eggs 0.927 other food 0.343
				Urban	staple food −0.073 vegetables and fruit 0.009 oil and fat 0.430 meat −0.045 eggs 0.545
Kaiyu Lyu, Xuemei Zhang, Li Xing and Chongshang Zhang	*International Conference of Agricultural Economists*	2015	Impact of Rising Food Prices on Food Consumption and Nutrition of China’s Rural Poor	Rural	staple food −0.096 vegetables and fruit 0.218 oil and fat 0.320 meat 0.126 eggs 0.447 other food 0.940 calorie 0.381 protein 0.023 fat 0.03 vitamin 0.167
Xiao Ye and J. Edward Taylor	*Economic Development and Cultural Change*	1995	The Impact of Income Growth on Farm Household Nutrient Intake: A Case Study of a Prosperous Rural Area in Northern China	Rural	staple food 0.462 vegetables and fruit 1.418 oil and fat1.074 meat 1.074 dairy products 1.124 calorie 1.26 protein 0.258 fat 0.216
Huang, Kuo S. Gale, Fred	*China Agricultural Economic Review*	2009	Food demand in China: Income, quality, and nutrient effects	Urban	staple food −0.065 vegetables and fruit 0.005 oil and fat 0.451 meat 0.275 eggs 0.536 other food 0.451 protein 0.065 fat 0.178 vitamin 0.147 minerals 0.243
Tian, Xu Yu, Xiaohua	*Frontiers of Economics in China*	2013	The demand for nutrients in China	Urban	protein 0.164 fat 0.273 vitamin 0.161 minerals 0.225
Zheng Zhihao	Beta Working Paper	2011	Household Food Demand by Income Category: Evidence from Household Survey Data in an Urban Chinese Province	Urban	staple food 0.801 vegetables and fruit 0.842 oil and fat 0.758 meat 0.885 eggs 1.246 other food 0.910 protein 0.903 fat 0.984 vitamin 0.986
Hovhannisyan, Vardges Mendis, Sachintha Bastian, Chris	*Agricultural Economics (United Kingdom)*	2019	An econometric analysis of demand for food quantity and quality in urban China	Urban	staple food 0.571 vegetables and fruit 0.624 oil and fat 0.69 meat 0.284 eggs 0.075 other food 0.677
Tong Han and Thomas I. Wahl	*Journal of Agricultural and Applied Economics*	1998	China’s Rural Household Demand for Fruit and Vegetables	Rural	staple food 1.092 vegetables and fruit 1.029 oil and fat 0.592 meat 0.462 dairy products 0.620
Brian W. Goulda, Hector J. Villarrealb	*Agricultural Economics*	2006	An assessment of the current structure of food demand in urban China	Urban	vegetables and fruit 0.950 oil and fat 0.850 meat 1.340 eggs 1.400 other food 0.960
Davis, John	*Applied Economics*	2008	Household Food Demand in Rural China	Rural	staple food 0.655 vegetables and fruit 0.445 meat 0.818 eggs 0.500 dairy products 0.520 other food 0.630
Christine Burggraf1, Lena Kuhn1, Qiran Zhao1, Thomas Glauben1, Ramona Teuber1	*Journal of Integrative Agriculture*	2014	Economic Growth and Nutrition Transition: An Empirical Analysis Comparing Demand Elasticities For Foods in China and Russia	Nation	vegetables and fruit 0.504 oil and fat 1.200 meat 0.500 eggs 1.38 dairy products 0.872 other food 0.911
Yang Gao and Zhihao Zheng	*China Agricultural Economic Review*	2020	Is nutritional status associated with income growth? Evidence from Chinese adults	Nation	protein 0.076 fat 0.112 vitamin 0.230 minerals 0.048

## References

[1] IEG Members (2021). Global Nutrition Report: The State of Global Nutrition. https://globalnutritionreport.org/reports/2021-global-nutrition-report/.

[2] UN (2019). Global Sustainable Development Report 2019: The Future Is Now—Science for Achieving Sustainable Development. https://sustainabledevelopment.un.org/gsdr2019.

[3] Cui T., Xi J., Tang C., Song J., He J., Brytek-Matera A. (2021). The Relationship between Music and Food Intake: A Systematic Review and Meta-Analysis. Nutrients.

[4] Yu X., Abler D. (2016). Matching Food with Mouths: A Statistical Explanation to the Abnormal Decline of per Capita Food Consumption in Rural China. Food Policy.

[5] FAO, IFAD, UNICEF, WFP, WHO (2020). In Brief to the State of Food Security and Nutrition in the World 2020. Transforming Food Systems for Affordable Healthy Diets.

[6] Huang Y., Tian X. (2019). Food Accessibility, Diversity of Agricultural Production and Dietary Pattern in Rural China. Food Policy.

[7] FAO, IFAD, WFP, WHO, UNICEF (2019). Food Security and Nutrition in the World Safeguarding Against Economic Slowdowns and Downturn.

[8] Arau S.J., Tirode F., Coin F., Pospiech H., Syva J.E., Stucki M., Hu U., Egly J., Wood R.D., Adamo A. (2017). Overweight Perception: Associations with Weight Control Goals, Attempts and Practices among Chinese Female College Students. Physiol. Behav..

[9] National Health Commitment, People’s Republic of China, Healthy Oral Action Program (2019–2025). http://www.gov.cn/xinwen/2019-02/16/content_5366239.htm.

[10] Paloma G.Y. Income Growth and Malnutrition in Africa: Is There a Need for Region- Specific Policies?. Proceedings of the 90th Annual Conference of the Agricultural Economics Society, University of Warwick.

[11] Ecker O., Comstock A. (2021). Income and Price Elasticities of Food Demand (E-FooD) Dataset: Documentation of Estimation Methodology.

[12] Gallet C.A. (2010). The Income Elasticity of Meat: A Meta-Analysis. Aust. J. Agric. Resour. Econ..

[13] Popkin B.M. (2014). Synthesis and Implications: China’s Nutrition Transition in the Context of Changes across Other Low- and Middle-Income Countries. Obes. Rev..

[14] Haen H., Klasen S., Qaim M. (2011). What Do We Really Know? Metrics for Food Insecurity and Undernutrition. Food Policy.

[15] Han T., Gail L., Cramer T.I.W. Rural Household Food Consumption in China: Evidence from the Rural Household Survey. Proceedings of the 1997 WAEA Meeting.

[16] Hovhannisyan V., Mendis S., Bastian C. (2019). An Econometric Analysis of Demand for Food Quantity and Quality in Urban China. Agric. Econ..

[17] Carter C.A., Zhong F. (1999). Rural Wheat Consumption in China. Am. J. Agric. Econ..

[18] Zheng Z., Henneberry S.R., Zhao Y., Gao Y. (2019). Predicting the Changes in the Structure of Food Demand in China. Agribusiness.

[19] Hanna K.L., Collins P.F. (2015). Relationship between Living Alone and Food and Nutrient Intake. Nutr. Rev..

[20] Liu H., Hu X. (2013). Effects of Income Growth on Nutritional Demand of Urban Residents in China. J. Agrotech. Econ..

[21] Tian X. (2013). The Demand for Nutrients in China. Front. Econ. China.

[22] Zhang C., Zhang X., Zhang X., Lyu K. (2014). Determination of Rural Poverty Line in China—A method based on Nutrition Perspective. Econ. Political Wkly..

[23] Nie P., Sousa-Poza A. (2016). A Fresh Look at Calorie-Income Elasticities in China. China Agric. Econ. Rev..

[24] Chen Y., Li G. (2018). Income Level, Aging and Nutritional Intake: A Study Based on Urban Household Data in Guangdong Province. South. Econ..

[25] Tian X., Yu X. (2015). Using Semiparametric Models to Study Nutrition Improvement and Dietary Change with Different Indices: The Case of China. Food Policy.

[26] You J., Imai K.S., Gaiha R. (2016). Declining Nutrient Intake in a Growing China: Does Household Heterogeneity Matter?. World Dev..

[27] Ogundari K., Abdulai A. (2013). Examining the Heterogeneity in Calorie—Income Elasticities: A Meta-Analysis. Food Policy.

[28] Santeramo F.G., Shabnam N. (2015). The Income-Elasticity of Calories, Macro- and Micro-Nutrients: What Is the Literature Telling Us?. Food Res. Int..

[29] Zhou D., Yu X. (2015). Calorie Elasticities with Income Dynamics: Evidence from the Submitted Article Calorie Elasticities with Income Dynamics: Evidence from the Literature. Appl. Econ. Perspect. Policy.

[30] Zamora J., Abraira V., Muriel A., Khan K., Coomarasamy A. (2006). Meta-DiSc: A Software for Meta-Analysis of Test Accuracy Data. BMC Med. Res. Methodol..

[31] Gallet C.A. (2007). The Demand for Alcohol: A Meta-Analysis of Elasticities. Aust. J. Agric. Resour. Econ..

[32] Chen D., Abler D., Zhou D., Yu X., Thompson W. (2016). Submitted Article A Meta-Analysis of Food Demand Elasticities for China. Appl. Econ. Perspect. Policy.

[33] Yu X. (2015). Meat Consumption in China and Its Impact on International Food Security: Status Quo, Trends, and Policies. J. Integr. Agric..

[34] De los Santos-Montero L.A., Bravo-Ureta B.E., von Cramon-Taubadel S., Hasiner E. (2020). The Performance of Natural Resource Management Interventions in Agriculture: Evidence from Alternative Meta-Regression Analyses. Ecol. Econ..

[35] Colen L., Melo P.C., Abdul-Salam Y., Roberts D., Mary S., Gomez Y., Paloma S. (2018). Income Elasticities for Food, Calories and Nutrients across Africa: A Meta-Analysis. Food Policy.

[36] Stanley T.D., Doucouliagos H. (2012). Meta-Regression Analysis in Economics and Business.

[37] Green R. (2013). The Effect of Rising Food Prices on Food Consumption: Systematic Review with Meta-Regression. BMJ.

[38] Salois M.J., Tiffin R., Balcombe K.G. (2012). Impact of Income on Nutrient Intakes: Implications for Undernourishment and Obesity. J. Dev. Stud..

[39] Zhou D., Yu X., Abler D., Chen D. (2020). Projecting Meat and Cereals Demand for China Based on a Meta-Analysis of Income Elasticities. China Econ. Rev..

[40] Gallet C.A., List J.A. (2003). Cigarette Demand: A Meta-Analysis of Elasticities. Health Econ..

[41] Tian X., Yu X. (2012). China Economic Review the Enigmas of TFP in China: A Meta-Analysis. China Econ. Rev..

[42] Taylor J.E. (1995). The Impact of Income Growth on Farm Household Nutrient Intake: A Case Study of a Prosperous Rural Area in Northern China. Econ. Dev. Cult. Chang..

[43] Ali M., Villa K.M., Joshi J. (2018). Health and Hunger: Nutrient Response to Income Depending on Caloric Availability in Nepal. Agric. Econ..

[44] Dolgopolova I., Teuber R. (2018). Consumers’ Willingness to Pay for Health Benefits in Food Products: A Meta-Analysis. Appl. Econ. Perspect. Policy.

[45] Jiang B., Davis J. (2007). Household Food Demand in Rural China. Appl. Econ..

[46] Zheng Z., Henneberry S.R. (2012). Estimating the Impacts of Rising Food Prices on Nutrient Intake in Urban China. China Econ. Rev..

[47] Yu X., Abler D. (2009). The Demand for Food Quality in Rural China. Am. J. Agric. Econ..

[48] Lyu K., Zhang X., Xing L., Zhang C. Impact of Rising Food Prices on Food Consumption and Nutrition of China’s Rural Poor. Proceedings of the International Association of Agricultural Economists 2015 Conference.

[49] Afshin A., Peñalvo J.L., Del Gobbo L., Silva J., Michaelson M., O’Flaherty M., Capewell S., Spiegelman D., Danaei G., Mozaffarian D. (2017). The Prospective Impact of Food Pricing on Improving Dietary Consumption: A Systematic Review and Meta-Analysis. PLoS ONE.

[50] Wu B., Shang X., Chen Y. (2021). Household Dairy Demand by Income Groups in an Urban Chinese Province: A Multistage Budgeting Approach. Agribusiness.

[51] Lewbel A. (1991). The Rank of Demand Systems: Theory and Nonparametric Estimation. Econometrica.

[52] Ogundari K., Bolarinwa O.D. (2018). Impact of Agricultural Innovation Adoption: A Meta-Analysis. Aust. J. Agric. Resour. Econ..

[53] Alinaghi N., Reed W.R. (2018). Meta-Analysis and Publication Bias: How Well Does the FAT-PET-PEESE Procedure Work?. Res. Synth. Methods.

[54] Egger M., Smith G.D., Schneider M., Minder C. (1997). Bias in Meta-Analysis Detected by a Simple, Graphical Test. BMJ.

[55] Stanley T.D. (2001). Wheat from Chaff: Meta-Analysis as Quantitative Literature Review. J. Econ. Perspect..

[56] Stanley T.D. (2008). Meta-Regression Methods for Detecting and Estimating Empirical Effects in the Presence of Publication Selection*. Oxf. Bull. Econ. Stat..

[57] Penn J.M., Hu W. (2018). Understanding Hypothetical Bias: An Enhanced Meta-Analysis. Am. J. Agric. Econ..

[58] Golden C.D., Koehn J.Z., Shepon A., Passarelli S., Free C.M., Viana D.F., Matthey H., Eurich J.G., Gephart J.A., Fluet-Chouinard E. (2021). Aquatic Foods to Nourish Nations. Nature.

[59] Gao Y., Zheng Z., Henneberry S.R. (2020). Is Nutritional Status Associated with Income Growth? Evidence from Chinese Adults. China Agric. Econ. Rev..

[60] Burggraf C., Kuhn L., Zhao Q., Glauben T., Teuber R. Economic Growth and Nutrition Transition: An Empirical Analysis Comparing Demand Elasticities for Foods in China and Russia Economic. Proceedings of the 2014 International Congress.

[61] Gong W., Liu A., Yao Y., Ma Y., Ding C., Song C., Yuan F., Zhang Y., Feng G., Chen Z. (2018). Nutrient Supplement Use among the Chinese Population: A Cross-Sectional Study of the 2010–2012 China Nutrition and Health Surveillance. Nutrients.

[62] Ren Y.J., Campos B.C., Loy J.P., Brosig S. (2019). Low-Income and Overweight in China: Evidence from a Life-Course Utility Model. J. Integr. Agric..

[63] Ortega D.L., Wang H.H., Wu L., Hong S.J. (2015). Retail Channel and Consumer Demand for Food Quality in China. China Econ. Rev..

[64] Hovhannisyan V., Devadoss S. (2020). Effects of Urbanization on Food Demand in China. Empir. Econ..

